# Gene-Specific Methylation Analysis in Thymomas of Patients with Myasthenia Gravis

**DOI:** 10.3390/ijms17122121

**Published:** 2016-12-16

**Authors:** Angela Lopomo, Roberta Ricciardi, Michelangelo Maestri, Anna De Rosa, Franca Melfi, Marco Lucchi, Alfredo Mussi, Fabio Coppedè, Lucia Migliore

**Affiliations:** 1 Department of Translational Research and New Technologies in Medicine and Surgery, Division of Medical Genetics, University of Pisa, Medical School, Via Roma 55, 56126 Pisa, Italy; angela.lopomo@student.unisi.it (A.L.); lucia.migliore@med.unipi.it (L.M.); 2 Department of Medical Biotechnologies, University of Siena, 53100 Siena, Italy; 3 Department of Clinical and Experimental Medicine, Neurology Unit, University of Pisa, Medical School, Via Roma 55, 56126 Pisa, Italy; r.ricciardi@ao-pisa.toscana.it (R.R.); michelangelo.mvg@tin.it (M.M.); annu.derosa@hotmail.it (A.D.R.); 4 Division of Thoracic Surgery, Cardiothoracic and Vascular Surgery Department, University of Pisa, Medical School, Via Roma 55, 56126 Pisa, Italy; franca.mamelfi@gmail.com (F.M.); marco.lucchi@unipi.it (M.L.); a.mussi@dc.med.unipi.it (A.M.)

**Keywords:** thymoma, myasthenia gravis, cancer, epigenetics, DNA methylation, *MTHFR*, *DNMT3A*

## Abstract

Thymomas are uncommon neoplasms that arise from epithelial cells of the thymus and are often associated with myasthenia gravis (MG), an autoimmune disease characterized by autoantibodies directed to different targets at the neuromuscular junction. Little is known, however, concerning epigenetic changes occurring in thymomas from MG individuals. To further address this issue, we analyzed DNA methylation levels of genes involved in one-carbon metabolism (*MTHFR*) and DNA methylation (*DNMT1*, *DNMT3A*, and *DNMT3B*) in blood, tumor tissue, and healthy thymic epithelial cells from MG patients that underwent a surgical resection of a thymic neoplasm. For the analyses we applied the methylation-sensitive high-resolution melting technique. Both *MTHFR* and *DNMT3A* promoters showed significantly higher methylation in tumor tissue with respect to blood, and *MTHFR* also showed significantly higher methylation levels in tumor tissue respect to healthy adjacent thymic epithelial cells. Both *DNMT1* and *DNMT3B* promoter regions were mostly hypomethylated in all the investigated tissues. The present study suggests that *MTHFR* methylation is increased in thymomas obtained from MG patients; furthermore, some degrees of methylation of the *DNMT3A* gene were observed in thymic tissue with respect to blood.

## 1. Introduction

Thymomas are uncommon neoplasms that arise from epithelial cells of the thymus and are often accompanied by non-neoplastic lymphocytic proliferation. On the basis of the morphology of epithelial cells and the lymphocyte to epithelial cell ratio, they are classified into five histological types, A, AB, B1, B2, and B3 [1]. The most widely used system for staging thymomas is the Masaoka–Koga staging system; according to this, thymomas are classified in stage I comprising encapsulated tumors, stages II and III showing direct local invasion, and stage IV showing metastatic spread [2]. Thymomas are associated with a wide variety of autoimmune diseases, and among these about 30%–40% of thymomas are associated with myasthenia gravis (MG), an autoimmune disease characterized by autoantibodies directed to different targets at the neuromuscular junction, such as acetylcholine receptor (AChR), muscle specific kinase (MuSK), and agrin-receptor low-density lipoprotein receptor related-protein 4 (LRP4) [3]. Almost all thymoma-associated MG (TAMG) patients have antibodies to the AChR; very rare exceptions have been seen in anti-MuSK+ or in double seronegative patients, while anti-LRP4 autoantibodies have not been reported [4]. The molecular events that characterize thymic neoplasms, including point mutations, chromosomal aberrations, and epigenetic modifications, such as changes in DNA methylation, have been described in the last few years [5]. Aberrant DNA methylation is the most widespread epigenetic alteration in carcinogenesis, and consists of the addition of a methyl group to cytosines, mainly in a CpG dinucleotide context, leading to gene silencing when occurring in the promoter region of a gene [6]. The reversibility of epigenetic changes, unlike genetic modifications, makes them a therapeutic target since, for instance, demethylating drugs can re-express genes silenced by methylation [7].

Concerning thymomas, the silencing of tumor suppressor genes, such as *FHIT*, *MLH1*, and *E-cad*, by DNA promoter methylation, has been described [8]. Aberrant methylation of other genes such as *MGMT*, *CDKN2A*, *HPP1*, and *DAP-K*, associated with the loss of protein expression, was observed [9]. Interestingly, promoter methylation of the *CDKN2A* gene was reported in up to 11% of thymomas and 25% of thymic carcinoma [10], while aberrant *MGMT* methylation and loss of its protein expression was more frequent in thymic carcinoma than in thymoma [11].

Folate metabolism plays an important role in the methylation process as it provides one-carbon units for both purine and pyrimidine base synthesis or for the formation of *S*-adenosylmethionine (SAM), the universal donor of methyl groups, thus playing an important role in DNA or RNA synthesis and repair, and in DNA methylation processes [12]. A key role in this metabolism is provided by methylenetetrahydrofolate reductase (MTHFR), the enzyme that catalyzes the reaction, which directs the provision of methyl groups donated from folate cofactors towards the remethylation of homocysteine to methionine; this last produces SAM, which is then used by DNA methyltransferases (DNMTs) that transfer the methyl group from SAM to the DNA. As a consequence, folate deficiency can disrupt DNA integrity and promote carcinogenesis [13]. DNMT1 is primarily involved in the maintenance of DNA methylation patterns during development and cell division, whereas DNMT3A and DNMT3B are the de novo methyltransferases that establish DNA methylation patterns during early development [14]. Given supportive evidence from the literature of the contributions of aberrant methylation and expression of *MTHFR* and *DNMTs*, such as *DNMT1*, *DNMT3A*, and *DNMT3B*, in other types of cancer, such as those of the breast, lung, and brain [15,16,17], we performed the present study to evaluate the methylation levels of these genes in thymomas of patients with MG.

## 2. Results

### 2.1. Methylation Levels among Tissues

Table 1 shows the mean methylation levels of the *MTHFR*, *DNMT3A*, *DNMT3B*, and *DNMT1* genes in blood and tumor tissue DNA of all TAMG patients. *MTHFR* shows higher methylation levels in tumor tissue respect to blood (*p* = 0.007), and *DNMT3A* shows about 10% promoter methylation in tumor tissue but is completely demethylated in blood (*p* < 0.0001). *DNMT3B* and *DNMT1* studied regions were largely hypomethylated both in blood and tumor tissue DNA.

**Figure 1:**
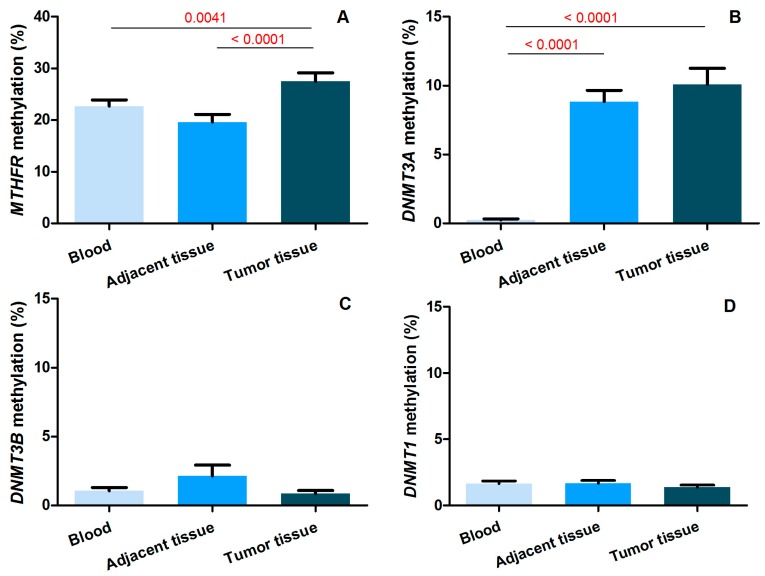
Methylation levels (%) of the studied genes ((**A**) *MTHFR*; (**B**) *DNMT3A*; (**C**) *DNMT3B*; and (**D**) *DNMT1*) in blood, tumor tissue and adjacent thymic tissue of thymoma-associated MG (TAMG) patients for whom adjacent tissue was available (*n* = 44). The significant *p*-values (in red) refer to *t*-tests for paired samples.

Figure 1 shows the mean methylation levels of the studied genes in blood, tumor tissue, and thymic epithelial tissue adjacent to the cancer, in the 44 patients for whom adjacent tissue was available. *MTHFR* shows higher methylation levels in tumor tissue with respect to blood (*p* = 0.0053) and to adjacent tissue (*p* < 0.001). *DNMT3A* shows higher methylation levels in tumor tissue and adjacent thymic tissue with respect to blood that results in them being completely demethylated (*p* < 0.001), but no significant difference was observed between thymomas and adjacent thymic tissue. The other two genes, *DNMT3B* and *DNMT1*, show very low methylation levels, no more than 2%, and no statistically significant difference was observed among the studied tissues. Age, gender, and clinico-pathological features had no effect on the mean methylation levels of the studied genes (Table S1).

### 2.2. Correlation of Methylation Levels among Tissues

**Figure 2:**
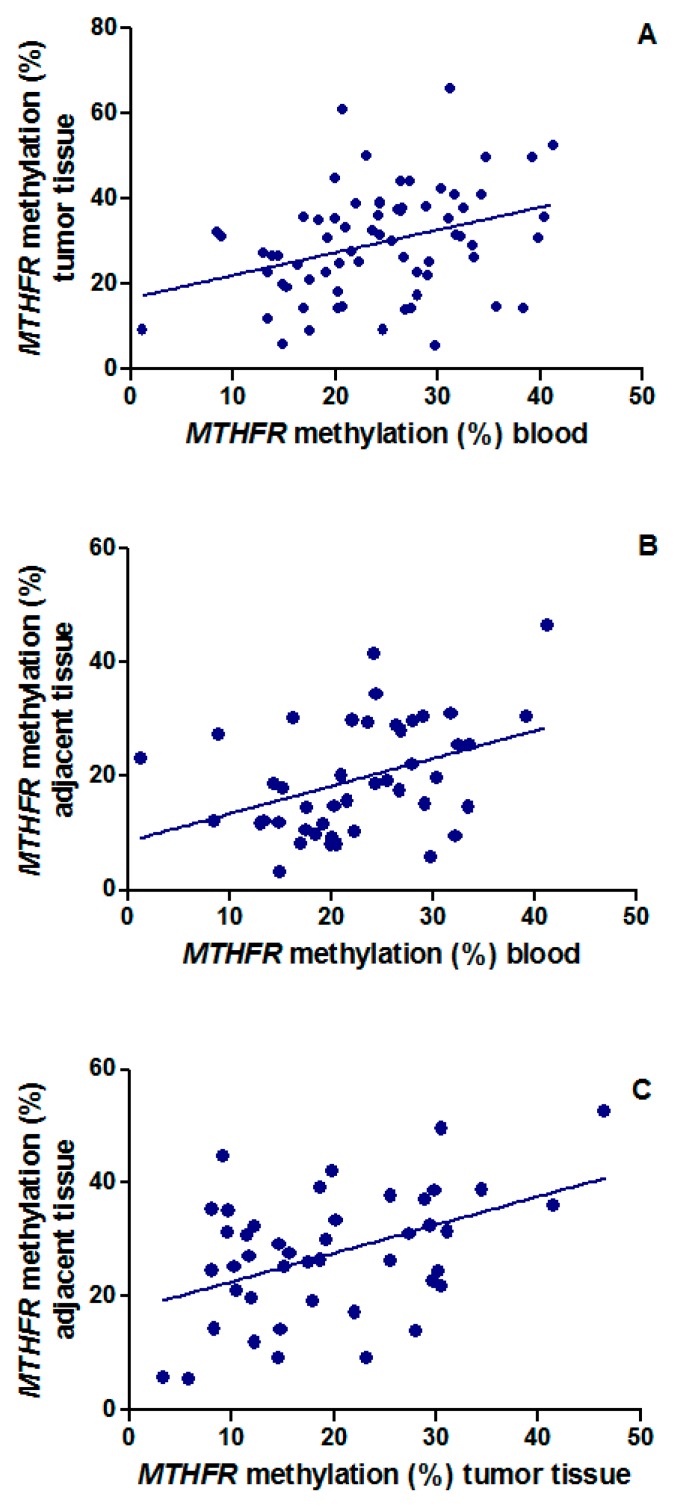
Correlation of *MTHFR* methylation levels (**A**) between tumor tissue and blood in the total population (*r* = 0.34; *p* = 0.004); (**B**) between adjacent tissue and blood (*r* = 0.40; *p* = 0.007); and (**C**) between tumor and adjacent thymic tissue (*r* = 0.45; *p* = 0.002) in the subgroup of 44 individuals with available thymic tissue adjacent to the tumor; *r* = Pearson’s correlation coefficient.

Interesting correlations between the *MTHFR* methylation levels among blood, tumor tissue, and thymic tissue adjacent to the tumor were observed (Figure 2A–C). In particular, correlation of *MTHFR* methylation levels between tumor tissue and blood was observed in the whole population (Figure 2A; *n* = 69; *p* = 0.004, *r* = 0.34) Moreover, correlations between blood and thymic tissue adjacent to the tumor (Figure 2B; *n* = 44; *p* = 0.007, *r* = 0.40) and between tumor tissue and adjacent thymic tissue (Figure 2C; *n* = 44; *p* = 0.002, *r* = 0.45) were observed.

## 3. Discussion

In the present study we investigated the methylation levels of genes involved in DNA methylation reactions, such as *DNMT1*, *DNMT3A*, and *DNMT3B*, and in one-carbon metabolism, *MTHFR*, in blood and tumor tissue DNA from 69 thymoma-associated myasthenia gravis patients; for 44 of them we also had thymic epithelial tissue adjacent to the tumor. We observed that *MTHFR* and *DNMT3A* promoters show different methylation levels between blood and tumor tissue; this data was observed in the total population composed of 69 patients as well as in the smaller subgroup of 44 patients for whom healthy thymic specimens were available. In the subgroup of 44 patients, different *MTHFR* methylation levels between the adjacent thymic tissue and the tumor tissue were noticed; in particular, tumor tissue showed on average higher methylation levels with respect to adjacent healthy tissue. For *DNMT3A*, higher methylation levels in the healthy tissue adjacent to a tumor were observed with respect to blood. Comparing the methylation levels of the studied genes in different tissues, statistically significant correlations of *MTHFR* promoter methylation levels between blood and tumor tissue, adjacent thymic tissue and blood, and tumor tissue and adjacent tissue were observed.

MTHFR is one of the major enzymes in folate metabolism since it catalyzes the irreversible conversion of 5,10-methylene THF (the methyl donor in the conversion of dUMP to dTMP) into 5-methyl THF, which remethylates homocysteine to methionine, necessary for the formation of *S*-adenosylmethionine, the universal donor of methyl groups; therefore, this key protein controls whether folate is partitioned towards DNA precursor synthesis or DNA methylation, and an alteration of this enzyme can interfere with the provision of methyl groups necessary for DNA methylation reactions. It is revealing that the *MTHFR* gene is regulated by promoter methylation and an increased *MTHFR* promoter methylation has been observed in several human diseases, such as cardiovascular and renal diseases [18,19], male infertility [20,21,22], and preeclampsia [23]. Moreover, it is also emerging that *MTHFR* hypermethylation could be involved in cancer formation; in fact, a correlation between *MTHFR* hypermethylation and lung cancer or cervical cancer lesions was observed [15,24].

In our cohort we observed higher *MTHFR* methylation levels in tumor tissue with respect to blood and adjacent thymic tissue, and we found a strong correlation between methylation levels in blood and tumor tissue. To our knowledge, this is the first time that *MTHFR* methylation has been analyzed in TAMG. We studied a CpG island previously associated with gene silencing; in fact, an inverse correlation was observed between the methylation levels of this region and gene expression levels, suggesting that hypermethylation of the *MTHFR* gene at the studied CpG sites may have a functional significance and may result in partial or complete silencing of the gene [15]. The silencing of *MTHFR* could cause a significant decrease in the global 5-methylcytosine content, leading to the activation of proto-oncogenes, as well as to global hypomethylation, as demonstrated in lung cancer by Vaissière and coworkers [15]. Furthermore, this can increase the availability of 5,10-methylene THF for the synthesis of thymidine and purine, causing the hyper-proliferation of cancerous cells. In this regard, it was suggested that *MTHFR* hypermethylation might confer a growth advantage to cancer cells and contribute to the cancer phenotype in tumors of the upper aero-digestive tract [25]. Moreover, *MTHFR* promoter methylation levels have been correlated with cancer risk factors and with markers of impaired folate metabolism, including tobacco smoking, low circulating folates and vitamin B12, high homocysteine levels, and increased chromosome instability [15,18,26,27].

This study revealed a clear involvement of *MTHFR* promoter methylation in TAMG, particularly the higher methylation levels in tumor tissue with respect to other tissues. These finding leads us to suppose that it could be involved in mechanisms associated with the development and progression of cancer, especially in the activation of proto-oncogenes. Obviously other studies need to further explore the significance of *MTHFR* methylation in TAMG, in order to better understand its pathogenic role in the onset of thymomas. The MTHFR protein is also required for the synthesis of DNA precursors, and impairments of this protein could contribute to cancer development by increasing the rate of point mutations and chromosome instability in rapidly dividing cells [12]. We observed a correlation among *MTHFR* promoter methylation levels in blood, thymic epithelial cells adjacent to the cancer, and thymoma cells that, albeit weak, was statistically significant. Such a correlation could suggest that *MTHFR* methylation levels in blood might reflect those in other tissues, and might represent a peripheral biomarker of the disease. In this regard, it was observed that females with increased *BRCA1* blood methylation have a 3.5-fold increased risk for early-onset breast cancer with histological features commonly seen in tumors arising in women with germline *BRCA1* mutations [28]. Similarly, glutathione *S*-transferase 1 (*GSTP1*) promoter methylation in peripheral DNA is regarded as a potential prognostic marker of prostate cancer [29]. However, as far as *MTHFR* promoter methylation in the blood is concerned, it should be noted that rather than being a specific marker of a given disease, it could represent a more general biomarker of increased genomic instability. For example, some studies suggest a correlation between hyperhomocysteinemia and *MTHFR* promoter methylation [26]; others have linked *MTHFR* promoter methylation in blood cells with markers of chromosome damage, such as an increased frequency of micronuclei [27] or alterations of LINE-1 methylation and stability [15], and there is also indication that *MTHFR* promoter methylation in blood DNA might reflect dietary B-group vitamin deficiency [18,30] or environmental exposure to cancerous agents, such as those deriving from tobacco smoking [15]. Collectively, those studies suggest that increased *MTHFR* promoter methylation in blood cells might be a more general marker of impaired one-carbon metabolism and genome instability, rather than a specific disease biomarker.

Another interesting question that still needs to be addressed is how an increased *MTHFR* promoter methylation could be linked to the regulation of autoimmune-related genes in TAMG patients. For example, it is known that the autoimmune regulator (*AIRE*) gene is hypomethylated in thymomas, and that both DNA and histone tail methylation marks regulate *AIRE* expression [31]. Therefore, we speculate that *MTHFR* methylation contributes to both DNA and histone tail methylation changes; however, this must be demonstrated in subsequent studies.

Regarding the DNMTs, the writers of DNA methylation reactions, in this study only *DNMT3A* showed increased methylation levels in tumor tissue, comparable to those found in adjacent thymic tissue, with respect to blood; the other two DNMTs genes, *DNMT1* and *DNMT3B*, resulted hypomethylated. The methylation of these genes was not yet investigated in thymomas but a previous study showed that they were overexpressed in the advanced stages of thymic epithelial tumors with respect to early stages [14].

A double role of de novo DNMTs in tumor stages, as oncogenes and tumor suppressor genes, was proposed. In healthy tissues, tumor suppressor genes are unmethylated and oncogenes are methylated. In early tumor stages, DNMTs seem to lead to methylation-associated repression of tumor suppressor genes and to promote tumor initiation, while in advanced tumor stages the downregulation of de novo DNMTs seems to be associated with promoter DNA hypomethylation of specific oncogenes and, consequently, would promote tumorigenesis [32].

In our cohort, *DNMT1* and *DNMT3B* do not show different methylation levels among different tissues, so we cannot hypothesize the involvement of the methylation of these genes in thymoma-associated myasthenia gravis pathogenesis.

Concerning the *DNMT3A* gene, it shows higher methylation levels in both cancer tissue and adjacent thymic tissue, where almost the same levels have been found with respect to blood, suggesting that its methylation is not connected exclusively with tumor events but already shows alterations in healthy tissue. Aberrant *DNMT3A* methylation was also observed in other cancers, such as in acute myeloid leukemia [16] and in breast cancer, where *DNMT3A* expression is associated with advanced stages [17]. Studies performed in mice with conditional knockout of Dnmt3a revealed a role for DNA methylation in mediating the self-renewal and differentiation of normal hematopoietic stem cells and leukemia stem cells [33]. A functional role of this gene in thymic epithelial tumors emerged from studies that focused on mutation analysis, revealing that *DNMT3A* is one the most frequently mutated genes in thymic carcinoma [34], and that the mutation p.G728D is associated with B3 thymomas [35]. Similarly, a SNP in the promoter of *DNMT3B*, namely −579G>T, was associated with TAMG [36], overall suggesting a contribution of de novo DNMTs in thymomas.

From the DNA promoter methylation analyses performed in this study, the involvement of *MTHFR* and *DNMT3A* methylation in thymoma-associated myasthenia gravis has been proven, even if other studies are needed to assess a potential pathogenic role of these genes in TAMG. Other limitations of the present study that need to be addressed in subsequent investigations include the analysis of gene expression levels of the studied genes in TAMG samples and a comparison study between TAMG samples and thymomas obtained from individuals without MG, which could help us to clarify whether the observed methylation changes are TAMG-specific or rather are common in thymomas.

## 4. Materials and Methods

### 4.1. Study Population

A total of 69 AChR positive patients (AChR+) with thymoma (TAMG) were recruited at the Myasthenia Clinic (Department of Neuroscience and Cardiac and Thoracic Department, Pisa University Hospital). The diagnosis of myasthenia gravis was made on the clinical symptoms of the patient together with the detection of positivity of AChR antibodies. Clinical stages of MG were assessed according to the Osserman classification. Demographic characteristics of the population are shown in Table 2. DNA samples were obtained from both the surgically resected tumor tissue of 69 patients (Table 2) and from the adjacent normal tissue available from 44 of them. An aliquot of blood in EDTA tubes was also collected from each patient and stored at −20 °C until assayed. Each patient gave informed written consent for genotype analysis before blood drawing. The study was conducted in accordance with the Declaration of Helsinki, and the protocol was approved by the Ethics Committee of the Pisa University Hospital (Protocol number 21302/2015).

### 4.2. Extraction of Genomic DNA

Genomic blood and tissue DNA was extracted using QIAmp DNA Mini Kit (Qiagen, Milan, Italy) according to the manufacturer’s instruction. The extracted DNA was quantified using a Nano Drop ND 2000c spectrophotometer (NanoDrop, Thermo Scientific, Wilmington, DE, USA).

### 4.3. Bisulfite Modification

Two hundred nanograms of DNA from each sample were treated with sodium bisulfite using the EpiTectH Bisulfite Kit (Qiagen) according to the manufacturer’s protocol. Sodium bisulfite treatment converts all unmethylated cytosines into uracil, while methylated cytosines are left unchanged.

### 4.4. Methylation-Sensitive–High-Resolution Melting (MS-HRM) Analysis

Promoter methylation was assessed by means of methylation-sensitive-high-resolution melting (MS-HRM) analysis in a CFX96 Real-Time PCR detection system (Bio-Rad, Milan, Italy). For the MS-HRM analysis we developed protocols according to the literature [37,38]. All analyses were run according to the following conditions: one cycle of 95 °C for 12 min, 60 cycles of 95 °C for 30 s, T_a_ for 30 s and 72 °C for 15 s; followed by an HRM step of 95 °C for 10 s and 50 °C for 1 min, 65 °C for 15 s, and continuous acquisition to 95 °C at one acquisition per 0.2 °C. PCR was performed in a final volume of 25 μL, containing 12.5 μL of master mix (Qiagen), 10 pmol of each primer, and 10 ng of bisulfite-modified DNA template. Each reaction was performed in duplicate. We analyzed 10% of the samples independently on separate occasions to verify the inter-assay variability and observed good reproducibility. Table 3 shows the conditions used for each gene, such as primers, annealing temperature, CpG sites, and amplicon length; for *MTHFR* and *DNMT3A* we used primers derived from the literature [15,16], while *DNMT1* and *DNMT3B* primers were designed by us using the Meth-primer program (available at http://www.urogene.org/methprimer). Fully methylated and unmethylated DNA (EpiTectH methylated and unmethylated human control DNA, bisulfite converted, Qiagen) were mixed to obtain the following ratios of methylation: 0%, 12.5%, 25%, 50%, 75%, and 100%. Standard curves with known methylation ratios were included in each assay and were used to deduce the methylation ratio of each blood, adjacent healthy tissue, and tumor tissue. Validation of the MS-HRM assays was performed by means of pyrosequencing, as detailed elsewhere [26]. In order to obtain single methylation percentage values from MS-HRM assays, we applied an interpolation method developed and described by us, which allowed us to obtain precise HRM methylation values comparable to those obtained by pyrosequencing [37].

### 4.5. Statistical Analysis

Differences in mean methylation levels among the three groups were evaluated with a Student’s *t*-test for paired samples. The effect of clinical and pathological features, such as onset of MG (early or late), MG Osserman classification, and thymoma histology, on mean methylation levels was assessed by means of analysis of variance (ANOVA), including age at sampling and gender as covariates. Linear regression analysis was performed to search for a correlation between age and methylation levels in the studied tissues, as well as to search for a correlation of *MTHFR* methylation levels among the different tissues. Analyses were performed with the STATGRAPHICS 5.1 Plus software package for Windows. Since we studied four different genes in our cohort, a Bonferroni’s correction for multiple comparisons was applied, and the cut-off *p*-value for considering a result to be statistically significant was set at 0.05/4 = 0.0125. The statistical power of the study was evaluated with the clinical tool and calculators for medical professionals, ClinCalc (Available at http://clincalc.com/Stats/Power.aspx). The sample size was chosen to have an a priori power of >80% to detect mean methylation differences of 5% or higher.

## 5. Conclusions

In conclusion, the present study revealed increased *MTHFR* promoter methylation in thymomas obtained from MG patients and some degrees of methylation of the *DNMT3A* gene in thymic tissue with respect to blood were observed. Other studies supported by gene expression analysis are needed to assess a potential pathogenic role of these genes in TAMG.

## Figures and Tables

**Table 1:** Methylation levels (%) of the studied genes in blood and tumor tissue of total thymoma-associated MG (TAMG) patients (*n* = 69). Blood and tumor tissues were taken from the same donors, and the *p*-value refers to a *t*-test for paired samples.

Gene	Blood (Mean ± SEM)	Tumor Tissue (Mean ± SEM)	*p*-Value
*MTHFR*	24.28 ± 0.99	29.66 ± 1.55	0.007
*DNMT3A*	0.35 ± 0.08	9.78 ± 0.91	<0.0001
*DNMT3B*	1.14 ± 0.24	0.95 ± 0.26	0.408
*DNMT1*	1.67 ± 0.12	1.43 ± 0.13	0.041

**Table 2:** Demographic and clinico-pathological characteristics of the studied population (TAMG: thymoma-associated myasthenia gravis, MG: myasthenia gravis, F: females, M: males, NS: no specified tumor histology).

No. TAMG Patients	Age ± SD	Gender (%)	MG Onset (%)	MG Osserman Classification (%)	Thymoma Histology (%)	Masaoka Stage (%)
69	55.5 ± 13.2	F: 37 (54) M: 32 (46)	<50 years: 26 (38) ≥50 years: 43 (62)	I: 7 (10.0)	A: 13 (18.8)	I: 7 (10)
				IIA: 11 (16.0)	AB: 13 (18.8)	IIa: 15 (22)
				IIB: 34 (49.5)	B1: 5 (7.2)	IIb: 29 (42)
				III: 13 (19.0)	B2: 23 (33.4)	III: 7 (10)
				IV: 3 (4.0)	B3: 8 (11.6)	IVa: 9 (13)
				V: 1 (1.5)	B2-B3: 5 (7.2)	NS: 2 (3)
				-	NS: 2 (3.0)	-

**Table 3:** Primer sequences and annealing temperatures (T_a_) used during MS-HRM analysis, amplicon length, number of CpG sites for each gene, accession number, and amplified region (* DMR2: differentially methylated region = upstream promoter region involved in the regulation of transcript 2 of *DNMT3A* gene, details are provided in [16]).

Gene	Primer Sequences	T_a_	Amplicon Length	CpG Sites	Accession Number	Amplified Region
*MTHFR*	F 5′-TTTTAATTTTTGTTTGGAGGGTAGT-3′	54°	155 bp	7	NM_005957.4	from +30 to +184 bp
	R 5′-AAAAAAACCACTTATCACCAAATTC-3′					
*DNMT1*	F 5′-GGTATCGTGTTTATTTTTTAGTAA-3′	52°	114 bp	9	NG_028016.3	from −106 to +8 bp
	R 5′-ACGAAACCAACCATACCCAA-3′					
*DNMT3A*	F 5′-GGTTTGGGTTTATTGTAGGAAGGTTATTAAGGT-3′	58°	199 bp	7	NM_153759.3	DMR2 *
	R 5′-AATCCAAAACCCCCCTATCACGAAA-3′					
*DNMT3B*	F 5′-TGGTGTTGTGTGATTATAGTGG-3′	55°	174 bp	6	NG_007290.1	from −397 to −223 bp
	R 5′-TCACCCTAAAAAATCAAAAACC-3′					

## Supplementary Materials

Supplementary materials can be found at www.mdpi.com/1422-0067/17/12/2121/s1.
