# Supplementary material for: Gene-Specific Methylation Analysis in Thymomas of Patients with Myasthenia Gravis
Source: Int J Mol Sci. 2016 Dec 16;17(12):2121. doi: 10.3390/ijms17122121 (PMC5187921; doi:10.3390/ijms17122121)
Supplement: Supplementary file 1 [file ijms-17-02121-s001.pdf]

Supplementary Materials: Gene-Specific Methylation Analysis in Thymomas of Patients with Myasthenia Gravis

Angela Lopomo, Roberta Ricciardi, Michelangelo Maestri, Anna De Rosa, Franca Melfi, Marco Lucchi, Alfredo Mussi, Fabio Coppedè and Lucia Migliore

Table S1. Correlation among age, gender, thymic histology, Masaoka classification, MG onset, MG Osserman classification, and methylation levels of the studied genes in blood tumor tissue of all TAMG patients (n = 69). The effect of clinical and pathological features on mean methylation levels was assessed by means of analysis of variance (ANOVA), including age at sampling and gender as covariates. Linear regression analysis was performed to search for a correlation between age and methylation levels in the blood and tumor tissue.

	Age	Gender	Histology	Masaoka	MG Onset	Osserman
MTHFR blood	$p = 0.65$; $r = -0.05$	$p = 0.93$	$p = 0.79$	$p = 0.59$	$p = 0.25$	$p = 0.35$
MTHFR tumor	$p = 0.98$; $r = 0.001$	$p = 0.95$	$p = 0.08$	$p = 0.68$	$p = 0.37$	$p = 0.58$
DNMT3A blood	$p = 0.17$; $r = -0.16$	$p = 0.96$	$p = 0.18$	$p = 0.29$	$p = 0.82$	$p = 0.84$
DNMT3A tumor	$p = 0.28$; $r = 0.13$	$p = 0.77$	$p = 0.32$	$p = 0.45$	$p = 0.72$	$p = 0.81$
DNMT3B blood	$p = 0.70$; $r = -0.05$	$p = 0.38$	$p = 0.52$	$p = 0.97$	$p = 0.75$	$p = 0.91$
DNMT3B tumor	$p = 0.11$; $r = 0.19$	$p = 0.68$	$p = 0.63$	$p = 0.83$	$p = 0.80$	$p = 0.56$
DNMT1 blood	$p = 0.72$; $r = -0.04$	$p = 0.85$	$p = 0.53$	$p = 0.24$	$p = 0.62$	$p = 0.48$
DNMT1 tumor	$p = 0.94$; $r = 0.008$	$p = 0.57$	$p = 0.56$	$p = 0.43$	$p = 0.14$	$p = 0.30$
